# Fibroblast Growth Factor Receptors (FGFRs) in Human Sperm: Expression, Functionality and Involvement in Motility Regulation

**DOI:** 10.1371/journal.pone.0127297

**Published:** 2015-05-13

**Authors:** Lucía Saucedo, Gabriela N. Buffa, Marina Rosso, Tomás Guillardoy, Adrian Góngora, María J. Munuce, Mónica H. Vazquez-Levin, Clara Marín-Briggiler

**Affiliations:** 1 Instituto de Biología y Medicina Experimental (IBYME), CONICET-FIBYME, Buenos Aires, Argentina; 2 Laboratorio de Medicina Reproductiva, Area de Bioquímica Clínica, Facultad de Ciencias Bioquímicas y Farmacéuticas, Universidad Nacional de Rosario, Rosario, Santa Fe, Argentina; 3 Reprolab, Sanatorio Británico de Rosario, Rosario, Santa Fe, Argentina; Universidad Nacional Autónoma de México, MEXICO

## Abstract

Fibroblast growth factors receptors (FGFRs) have been widely characterized in somatic cells, but there is scarce evidence of their expression and function in mammalian gametes. The objective of the present study was to evaluate the expression of FGFRs in human male germ cells, to determine sperm FGFR activation by the FGF2 ligand and their participation in the regulation of sperm motility. The expression of FGFR1, 2, 3 and 4 mRNAs and proteins in human testis and localization of these receptors in germ cells of the seminiferous epithelium was demonstrated. In ejaculated sperm, FGFRs were localized to the acrosomal region and flagellum. Sperm exposure to FGF2 caused an increase in flagellar FGFR phosphorylation and activation of extracellular signal-regulated kinase (ERK) and protein kinase B (PKB or Akt) signaling pathways. Incubation with FGF2 led to a significant increase in the percentage of total and progressive sperm motility, as well as in sperm kinematics. All responses were prevented by sperm preincubation with BGJ398, a specific inhibitor of FGFR tyrosine kinase activity. In addition to confirming the expression of FGFRs in germ cells of the human testis, our study describes for the first time the presence, localization and functionality of human sperm FGFRs, and provides evidence of the beneficial effect of FGF2 upon sperm motility.

## Introduction

Fibroblast growth factors (FGFs) constitute a family of 17–34 kDa proteins, being FGF2 the best-characterized member of this family [1, 2]. FGFs bind to specific receptors (FGFRs) composed of 3 extracellular immunoglobulin-like domains, a single transmembrane domain, and 2 highly conserved cytoplasmic domains with tyrosine kinase activity. Among FGFRs, the most studied are FGFR1, FGFR2, FGFR3 and FGFR4 [3, 4]. Transcripts coding the extracellular domains of FGFR1, FGFR2 and FGFR3 are subjected to alternative splicing, giving rise to 2 or 3 receptor isoforms (IIIa, IIIb and IIIc) with specific tissue expression and different ligand binding properties [5]. In particular, FGF2 has been shown to bind with high affinity to FGFR1 IIIb and IIIc, FGFR2 IIIc, FGFR3 IIIc and FGFR4, but not to other FGFR isoforms [6]. Interaction of FGFs with heparin or heparan sulfate proteoglycans allows their binding to FGFRs, triggering receptor dimerization and phosphorylation [7]. Activation of FGFRs leads to the activation of the Ras/mitogen activated protein kinase (MAPK) or extracellular signal-regulated kinase (ERK) pathway as well as the phosphatidylinositol 3-kinase (PI3K)/protein kinase B (PKB or Akt) signaling pathway. In somatic cells, components of these signal transduction cascades translocate to the nucleus and phosphorylate specific transcription factors, inducing the expression of FGF-target genes [8].

FGF and FGFR expression has been reported in multiple tissues [9, 10] and both, ligands and receptors, have been implicated in cell proliferation, differentiation, adhesion, survival, apoptosis, and motility. This system has been related to normal tissue maintenance, repair and regulation as well as to tumor progression [11, 12]. In the female reproductive tract, FGFs and FGFRs have been involved in folliculogenesis, embryo implantation and development [13, 14]. Components of the FGF/FGFR pathway have also been found in tissues of the male reproductive tract from several species [6, 15, 16]. Transgenic mice expressing a dominant-negative variant of FGFR1 in the male haploid germ cells are subfertile, show diminished daily sperm output and reduced ability to undergo cellular changes associated with sperm capacitation [17], suggesting that this system has a relevant role in spermatogenesis/spermiogenesis and in the regulation of sperm physiology. Contrasting, a recent study reported that germ cell-specific FGFR1 or FGFR2 mutant mice have normal spermatogenesis and fertility, possibly due to compensatory mechanisms exerted by other FGFRs [18]. However, until the present time there are no reports on the FGFR expression and function in the human sperm.

The aim of the present study was to evaluate the expression and localization of FGFRs in human testis and sperm cells, to determine their activation in the male gamete in response to the FGF2 ligand, and to analyze the participation of the FGF/FGFR system in the regulation of sperm motility.

## Materials and Methods

### Ethics statement

All human samples (testicular tissue and sperm) used in the study were obtained under donors’ written consent, and protocols were approved by the Ethics Committee from the Instituto de Biología y Medicina Experimental, Buenos Aires, Argentina (Ref: CE 010-2/2013) and the Sanatorio Británico, Rosario, Argentina (Ref: 06-25-2013).

### Reagents and antibodies

All reagents were of tissue culture grade and molecular biology quality, purchased from Sigma Chemical Co. (St. Louis, MO, USA), GE-Amersham Pharmacia (Piscataway, NJ, USA), Thermo-Life Technologies (Carlsbad, CA, USA) and Qiagen (Hilden, Germany), unless indicated.

The following polyclonal antibodies and their corresponding blocking peptides (P) were used: anti FGFR1 (sc-121 and sc-121 P), anti FGFR2 (sc-122 and sc-122 P), anti FGFR3 (sc-123 and sc-123 P) and anti FGFR4 (sc-9006) (Santa Cruz Biotechnology Inc., Santa Cruz, CA, USA). Other antibodies used were: anti pFGFR Tyr653/654 (#3476, Cell Signaling Technology, Inc., Beverley, MA, USA), anti pERK (sc-7383, Santa Cruz, and #4370, Cell Signaling), anti ERK (sc-94, Santa Cruz), anti pAkt Ser473 (sc-7985, Santa Cruz, and #4060, Cell Signaling), anti Akt (#4691, Cell Signaling), rabbit immunoglobulin G (IgG) (Sigma), horse-radish peroxidase (HRP)-conjugated anti-rabbit IgG (Sigma), Cy3-conjugated anti-rabbit IgG and FITC-conjugated anti-mouse IgG (Chemicon-Millipore, Billerica, MA, USA) and anti-rabbit IgG (Sigma).

The FGF2 was produced in a bacterial expression system (gently provided by Dr. Baldi, IBYME), and was previously shown to activate FGFRs in breast cancer cells [19]. BGJ398 (NVP-BGJ398, Selleck Chemicals LLC, Houston, TX, USA), a selective inhibitor of FGFR tyrosine kinase activity [20], was used when indicated.

### Testicular tissue and cells

Human testicular tissues were obtained from adult patients undergoing orchiectomy as treatment for prostatic carcinoma, and not receiving any hormonal treatment prior to surgery.

To study the expression and functionality of FGFRs in the human sperm, semen samples from normozoospermic volunteers [21] were used. After complete liquefaction, highly motile sperm were recovered using the swim-up procedure [21]. Sperm suspensions were diluted with Biggers-Whitten-Whittingham medium (BWW; [21]) containing 0.3% bovine serum albumin (BSA), centrifuged at 300*g* for 10 min, resuspended in the same medium, and cells were either immediately processed or incubated at 37°C, 5% CO_2_ in air. The effect of FGF2 on sperm motility was analyzed using semen samples from individuals attending an andrology laboratory; only samples with less than 1 x 10^6^ round cells/ml were included. Seminal plasma was removed by centrifugation and sperm were resuspended in BWW supplemented with BSA, at a concentration of 20 x 10^6^ cells/ml.

MCF7 human breast cancer cells, which express FGFRs [22], were cultured following the supplier’s recommendations (ATCC, Manassas, VA, USA) as described [23], and were used as control in FGFR messenger RNA (mRNA) and protein expression studies.

### Tissue and cell RNA analysis

Total RNA from MCF7 cells, human testis and sperm (from 3 different donors) was isolated using the Trizol reagent (Thermo-Life) following the protocol suggested by the manufacturer. Complementary DNA (cDNA) was synthesized and subjected to a standard end-point PCR followed by electrophoresis in 3% agarose gels as previously described [24]. The following primers were used: for FGFR1 forward 5´-AGAGGACAATGTGATGAAGATA-3´ and reverse 5´-GGTCAAATAATGCCTCGGGT-3´, for FGFR2 forward 5´-AACGGGAAGGAGTTTAAGCA-3´ and reverse 5´-CTTGTCAGATGGGACCACAC-3´ [25], for FGFR3 forward 5´-AGGCCATCGGCATTGACA-3´ and reverse 5´-GCATCGTCTTTCAGCATCTTCAC-3´ [25], for FGFR4 forward 5´-CGCGGCGTCCACCACATT-3´ and reverse 5´-GTGTGTACACCCGGTCAAAC-3´. The expected sizes of the fragments were: 128 bp for FGFR1, 99 bp for FGFR2, 73 bp for FGFR3, and 100 bp for FGFR4. All samples were analyzed in triplicates; negative controls omitting the reverse transcriptase (RT Control) and the template (PCR Control) were included. Molecular weight markers were included in all electrophoretic runs.

### Protein extracts, SDS-PAGE and Western immunoblotting

Protein extracts of MCF7 cells, human testis and sperm were obtained as described [26]. Extracted proteins were placed in Laemmli sample buffer containing 5% 2-mercaptoethanol, boiled for 10 min, and subjected to SDS-PAGE in 8% polyacrylamide gels and Western immunoblotting as detailed [26]. Anti FGFR antibodies or rabbit IgG were used at 2 μg/ml. To determine antibody specificities, anti FGFRs were preincubated with the corresponding blocking peptides at a 5x concentration following the supplier’s recommendations. The reactive bands were detected by enhanced chemiluminiscence (ECL kit, Amersham) using standard procedures.

### Immunohistochemistry

Small portions of human testis were fixed and processed as previously described [24], using anti FGFR antibodies or rabbit IgG (4 μg/ml) and the LSAB + System HRP kit (K0690, Dako, Carpinteria, CA, USA). Antibody specificity was confirmed by antibody preincubation with the corresponding blocking peptide. Specimens were counterstained with hematoxylin, dehydrated and mounted. Sections were evaluated at x600 magnification using an Eclipse E800 microscope (Nikon Instruments Inc., Tokyo, Japan).

### Immunocytochemistry

Sperm were fixed and processed as described [26], using anti FGFR antibodies, rabbit IgG (20 μg/ml) or the anti FGFR antibodies previously incubated with the blocking peptides, and Cy3-conjugated secondary antibody. To assess the sperm acrosomal status, cells were stained with the lectin *Pisum sativum* agglutinin labeled with FITC (FITC-PSA; 50 μg/ml in PBS). A green fluorescent signal on the acrosomal region was indicative of an intact acrosome. At least 200 sperm in duplicate samples were evaluated using a Nikon fluorescence microscope (Nikon Instruments Inc., Melville, NY, USA). Images were acquired with a Nikon laser confocal microscope C1, using an objective PlanApo 60x/1.40 oil, excitation/emission: 488 nm/515-530 nm and 544 nm/570 LP.

### Assessment of sperm FGFR functionality

To analyze the functionality of sperm FGFRs, cells were incubated for 4 h and FGF2 (0–100 ng/ml) was added during the last 15 min of incubation, following a procedure previously described [27]. When indicated, BGJ398 (0.1 μM) was added 15 min prior to FGF2. Cells were processed for immunocytochemical analysis or protein extraction as detailed below.

Immunocytochemical analysis was performed using pFGFR (1:50), pERK (sc-7383; 20 μg/ml) or pAkt (sc-7985; 10 μg/ml) as primary antibodies and FITC-conjugated secondary antibodies; nuclei were stained with propidium iodide. Both fluorescence signals were recorded. The percentage of stained cells was calculated as (# of sperm with FITC-stained flagellum / # of sperm stained nuclei) x 100.

To obtain sperm protein extracts, sperm were diluted with PBS containing protease inhibitors, followed by centrifugation at 400*g* for 10 min. Pellets were incubated with RIPA buffer (50 mM Tris-HCl pH 7.4, 150 mM NaCl, 0.1% SDS, 1% Triton X-100, 1% NP-40, 0.5% sodium deoxycholate) supplemented with phosphatase and protease inhibitors, maintained on ice for 20 min and centrifuged at 14000*g* for 10 min. Protein extracts from 30 x 10^6^ cells were resuspended in Laemmli sample buffer and further processed for SDS-PAGE in 8% polyacrylamide gels and Western immunoblotting using anti pERK (#4370; 1:100), ERK (sc-94; 0.1 μg/ml), pAkt (#4060; 1:100) and Akt (#4691; 1:2000) antibodies. Densitometric analysis of the bands was performed using the ImageJ densitometry software 1.48 v (National Institutes of Health, Bethesda, MD, USA). The intensity values of pERK were normalized to those of ERK and of pAkt to those of Akt of the same loading; values of the control condition were considered as 1.

### Computer-assisted sperm analysis

To analyze the effect of FGF2 on sperm motility, samples were incubated for 2 h with FGF2 in the presence or in the absence of 0.1 μM BGJ398. Sperm motility parameters were evaluated using the Integrated Sperm Analysis System ISAS v1 (Proiser R&D, Valencia, Spain) that analyzes 25 frames per second (s) (total time analyzed 1 s). A microscope with a temperature-controlled stage was used to maintain sperm at constant 37°C during motility assessment. For each sample, at least 5 microscopic fields were analyzed and more than 300 motile sperm were evaluated. Motility parameters were measured and sperm motility was classified in Grade a (VAP ≥ 35 μm/s; STR ≥ 80%), Grade b (35 μm/s > VAP ≥ 10 μm/s), Grade c (10 μm/s > VAP ≥ 4 μm/s) or Grade d (VAP < 4 μm/s). Percentages of total (Grade a + b + c) and progressive (Grade a + b) motility were recorded.

### Statistical analysis

Data were expressed as mean ± standard error of the mean (SEM). To assume normal distribution, percentages were expressed as ratios and subjected to the arcsine square root transformation. Results were compared by one-way ANOVA and the Dunnett´s multiple comparison test. Statistical analyses were carried out using the GraphPad InStat program (GraphPad Software, San Diego, CA, USA).

## Results

### Expression and localization of FGFRs in human testis

**Fig 1:**
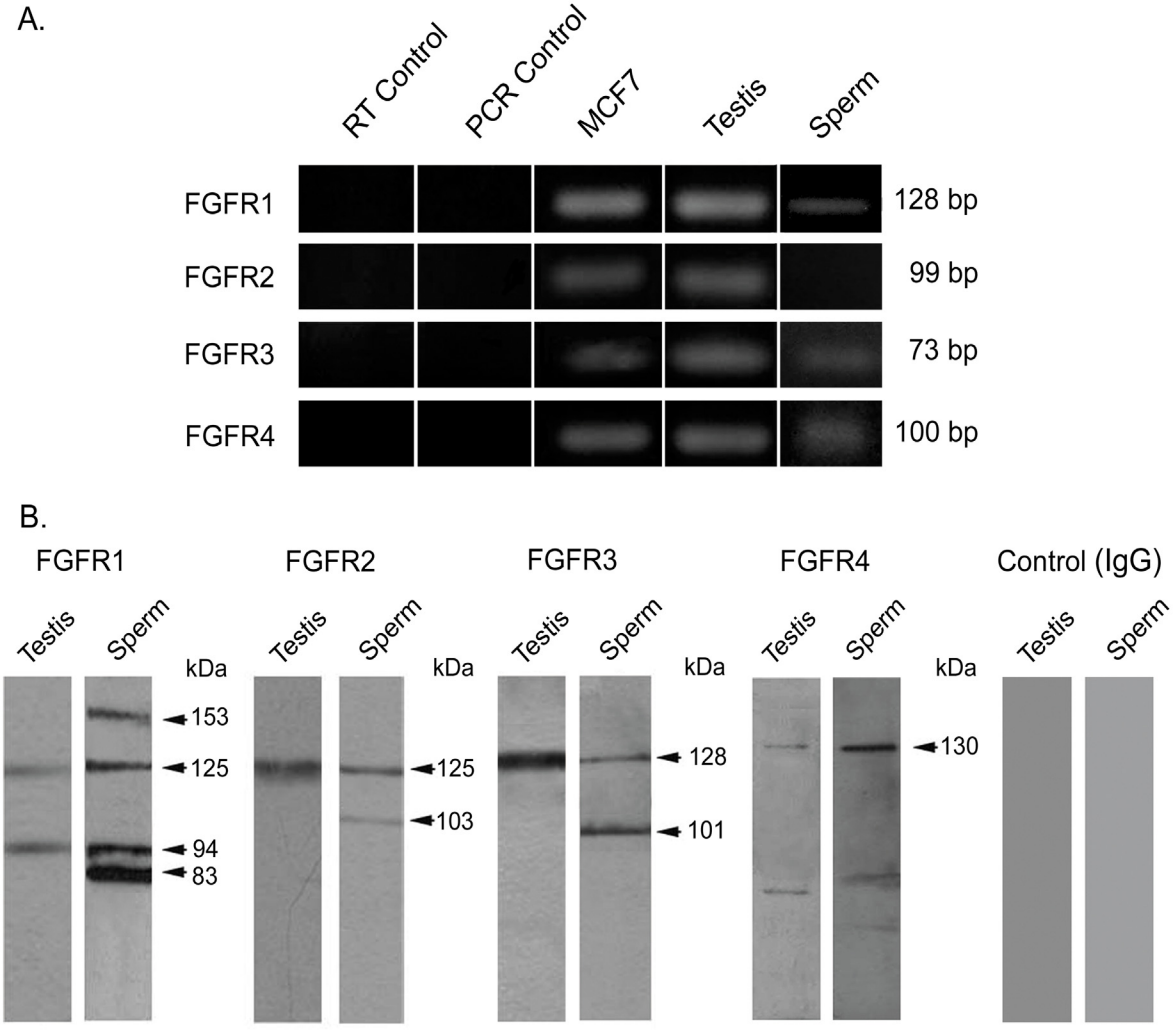
Expression of FGFRs in human testis and sperm. (**A**) Messenger RNA expression of testicular and sperm FGFRs assessed by RT-PCR. Messenger RNA extracted from MCF7 cells served as positive controls; negative controls without reverse transcriptase (RT Control) and without template (PCR Control) are shown. The amplicon sizes are indicated on the right. (**B**) Detection of testis and sperm FGFR protein forms using Western immunoblotting. Protein extracts from human testis and sperm were subjected to SDS-PAGE and Western immunoblotting using anti FGFR antibodies or rabbit IgG as control. The estimated molecular weights of the protein bands are indicated on the right. The experiments were performed at least 3 times obtaining similar results. Typical results are shown.

Expression of FGFR1, FGFR2, FGFR3 and FGFR4 mRNA was evaluated in human testis by RT-PCR, using MCF7 cells as control. Amplicons of the expected molecular size, corresponding to the 4 FGFRs, were detected in the reproductive tissue (Fig 1A). Amplification fragments were specific, since no signal was observed in the RT and PCR controls.

Western immunoblotting studies showed the presence of FGFR protein forms in human testis (Fig 1B). Testicular protein extracts developed with anti FGFR1 depicted bands of approximately 125 kDa and 94 kDa. Protein bands of 125, 128 and 130 kDa were detected with anti FGFR2, FGFR3 and FGFR4, respectively. These protein bands were similar to those found in MCF7, although some additional bands were obtained in these cell protein extracts (S1 Fig). No bands were detected when rabbit IgG was used in replacement of the primary antibodies (Fig 1B) or when the antibodies were preincubated with the corresponding blocking peptides (S1 Fig).

**Fig 2:**
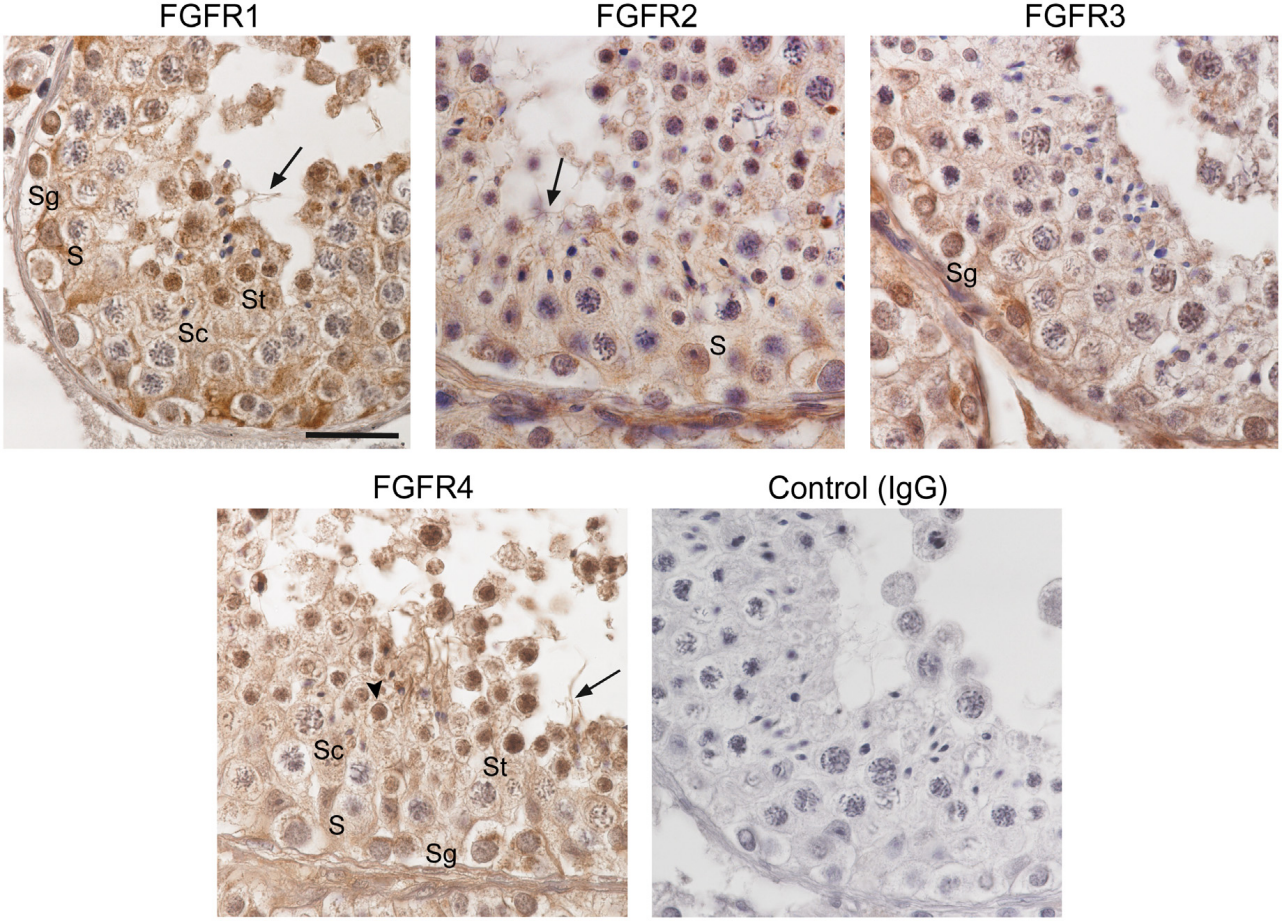
Localization of FGFRs in human seminiferous epithelium. Immunohistochemical analysis of FGFRs in human testis using anti FGFR antibodies; rabbit IgG was included as control. The specimens were counterstained with hematoxylin. S: Sertoli cell, Sg: spermatogonia, Sc: spermatocyte, St: spermatid. Arrows indicate immunoreactivity for FGFRs in the flagellum of elongating/elongated spermatids and the arrow head indicates FGFR4 immunoreactivity in spermatid acrosome. Bar: 20 μm.

Immunohistochemical analysis of human testis sections with anti FGFR1 showed staining in the nuclei and cytoplasm of Sertoli cells, spermatogonia and spermatids, as well as in the cytoplasm and flagellum of elongating/elongated spermatids. Moreover, a weak immunoreactivity for FGFR1 was observed in the nucleus of meiotic spermatocytes (Fig 2). With anti FGFR2, immunoreaction was detected mainly in the nucleus of Sertoli cells, in tubular walls and in testicular microvessels, but also in the cytoplasm and flagellum of elongating/elongated spermatids. Immunoreactivity for FGFR3 was found mainly in spermatogonia. FGFR4 immunolocalized to the nuclei and cytoplasm of Sertoli and all germ cells, and a strong staining was observed in the nucleus and acrosome of round spermatids, as well as in the cytoplasm and flagellum of elongating/elongated spermatids (Fig 2). The stainings were specific, since no immunoreaction was observed when rabbit IgG was used (Fig 2) or when the antibodies were preincubated with the blocking peptides (S2 Fig).

### Expression and localization of FGFRs in human ejaculated sperm

Sperm mRNA analysis led to the specific detection of amplicons for FGFR1, FGFR3 and FGFR4 (Fig 1A). Regarding protein expression, Western immunoblotting of sperm extracts using anti FGFR1 showed protein forms of 153 and 125 kDa, as well as other bands of lower molecular weight (94 and 83 kDa). Protein bands of 125 and 103 kDa were found with anti FGFR2 and of 128 and 101 kDa with anti FGFR3. A main protein form of 130 kDa was detected with anti FGFR4 (Fig 1B). All these bands were specific, as no signal was obtained using rabbit IgG in replacement of the primary antibodies (Fig 1B) or antibodies preincubated with blocking peptides (S1 Fig).

**Fig 3:**
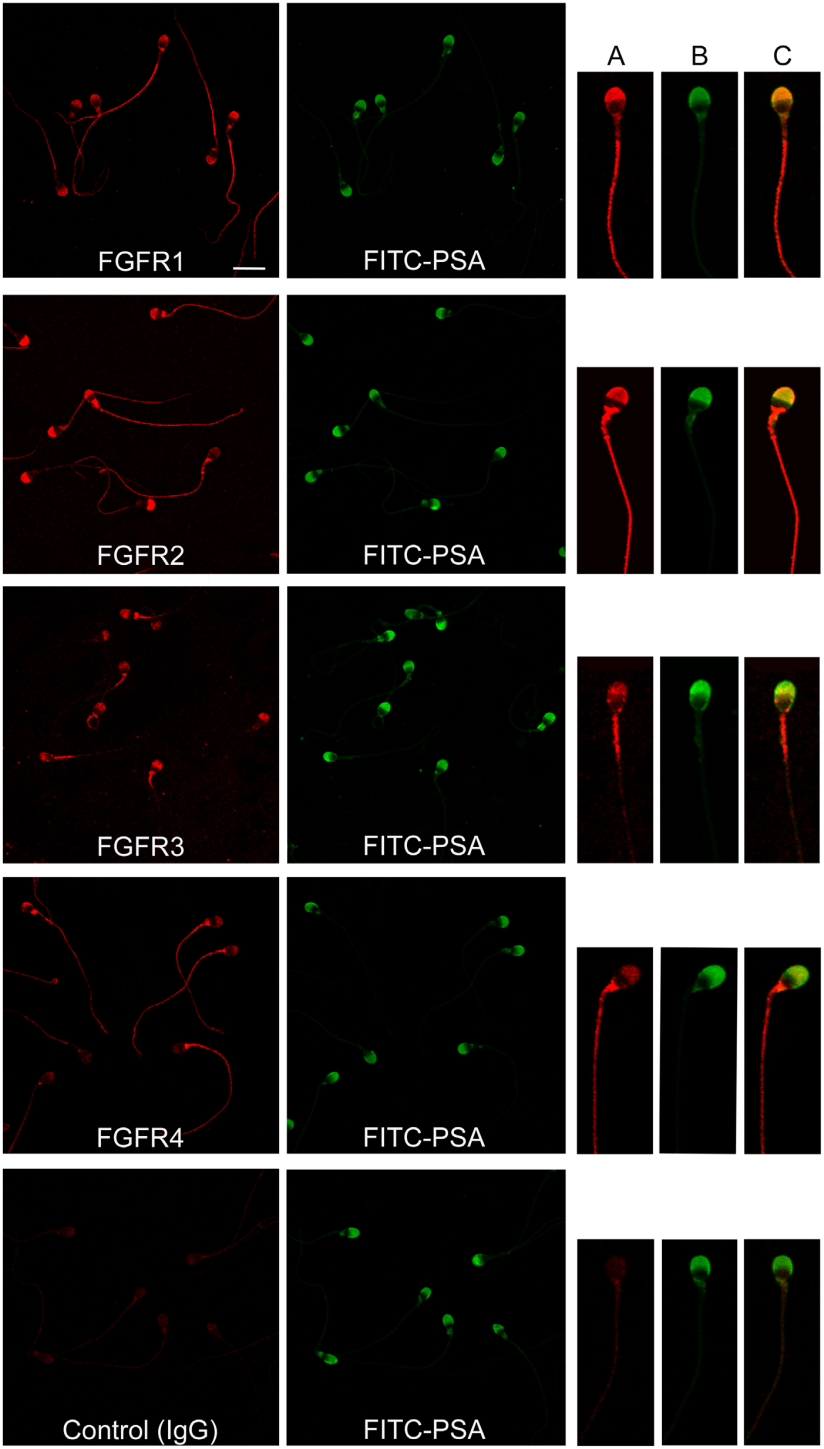
Localization of FGFRs in human sperm. Sperm cells were stained with anti FGFR1, FGFR2, FGFR3 and FGFR4 or rabbit IgG and a secondary antibody labeled with Cy3. The corresponding fields stained with FITC-PSA to assess acrosomal status are shown. Bar: 10 μm. On the right, a representative image of individual sperm is depicted; (**A**) sperm stained with anti FGFR antibody and Cy3-conjugated secondary antibody, (**B**) FITC-PSA, (**C**) merge.

To determine FGFR localization in human sperm, immunofluorescence analysis with anti FGFR antibodies was carried out (Fig 3). In all sperm suspensions evaluated, over 90% of the cells were classified as acrosome intact by means of the FITC-PSA staining. FGFR1 was localized to the principal piece of the flagellum in 100% of the cells and to the acrosomal region in 81 ± 2% of the sperm (n = 8). FGFR2 was detected along the flagellum of all cells and in the acrosomal region in 84 ± 1% of the cells (n = 7); in addition, a strong signal in the neck was found in some cells. Sperm stained with anti FGFR3 showed label in the midpiece of the flagellum, with a faint signal in the principal piece, and in the acrosomal region (80 ± 4%) (n = 8). All cells stained with anti FGFR4 depicted an intense signal along the flagellum and 69 ± 10% of the sperm showed label in the acrosomal region (n = 5). FGFR immunodetection was specific, since no staining was observed when primary antibodies were replaced by rabbit IgG (Fig 3) or when the antibodies were blocked with the respective peptides (S3 Fig).

### Activation of sperm FGFRs and FGFR-related intracellular pathways

**Fig 4:**
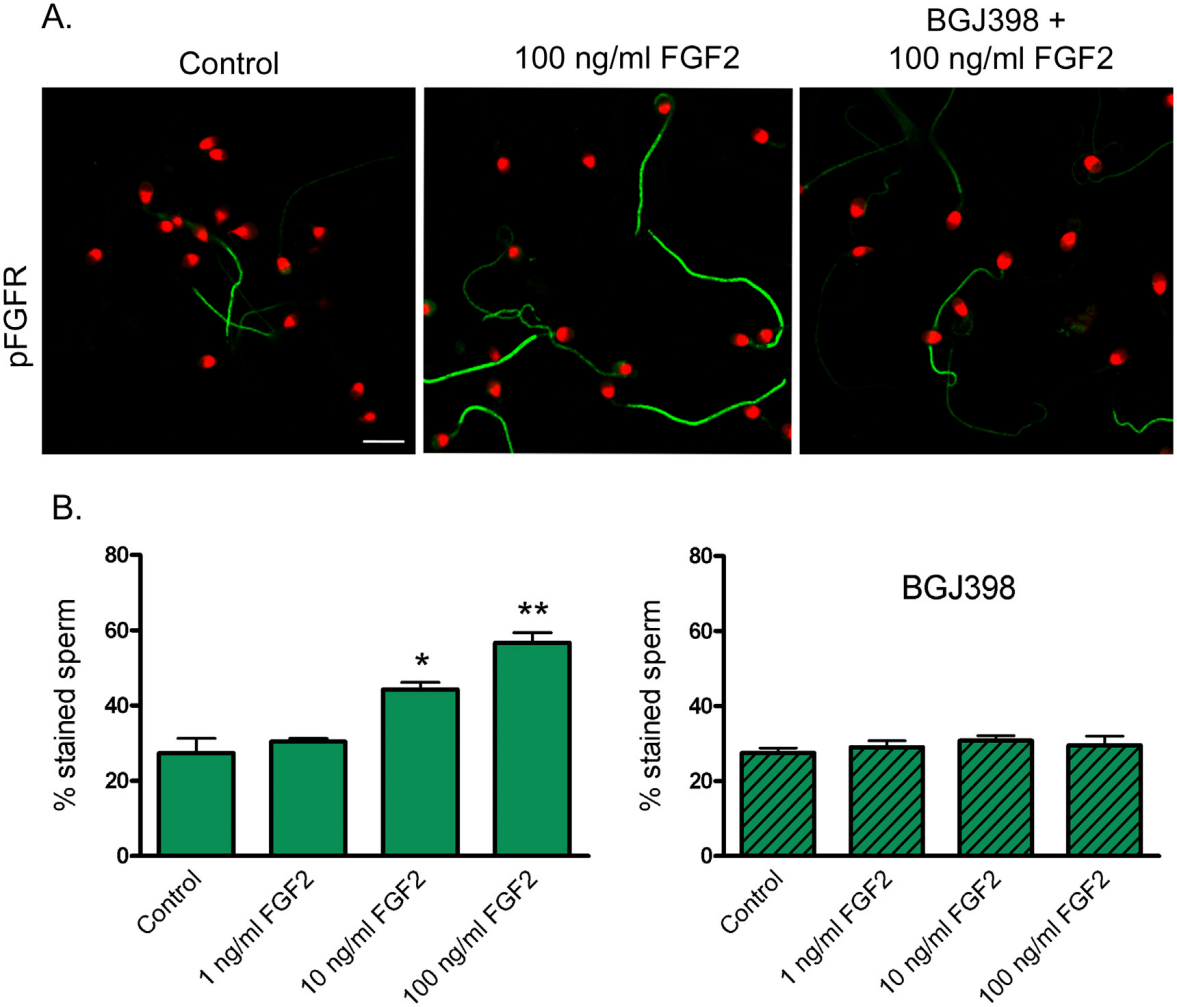
Activation of sperm FGFRs in response to FGF2. Localization of sperm pFGFRs by immunocytochemistry. Sperm were incubated for 4 h and exposed to FGF2 (0, 1, 10 and 100 ng/ml) for the last 15 min. In some aliquots, sperm were incubated for 15 min with BGJ398 (0.1 μM) before the addition of FGF2. Sperm were processed for immunocytochemistry, stained with anti pFGFR and FITC-conjugated secondary antibody; nuclei were stained with propidium iodide. (**A**) pFGFR immunolocalization in sperm incubated in the absence of FGF2 (Control), with 100 ng/ml FGF2, and with BGJ398 + 100 ng/ml FGF2. Bar: 10 μm. (**B**) Percentage of sperm cells stained with anti pFGFR antibody after exposure to different concentrations of FGF2 in the absence (**left**) or presence of BGJ398 (**right**). Results are expressed as mean ± SEM, n = 4. * *P* < 0.05; ** *P* < 0.01 compared with Control.

To analyze the activation of sperm FGFRs, cells were exposed to FGF2 and FGFR phosphorylation was evaluated by immunocytochemistry. When incubated for 4 h in the absence of FGF2, 27 ± 4% of the sperm showed phosphorylation of the flagellar FGFRs, depicting differences in signal intensity among the cells (Fig 4). Sperm exposure to 10 and 100 ng/ml FGF2 led to a significant increase (*P* < 0.05) in the percentage of stained cells (44 ± 2% and 57 ± 3%, for 10 and 100 ng/ml FGF2, respectively). The addition of the inhibitor BGJ398 suppressed such response (Fig 4).

**Fig 5:**
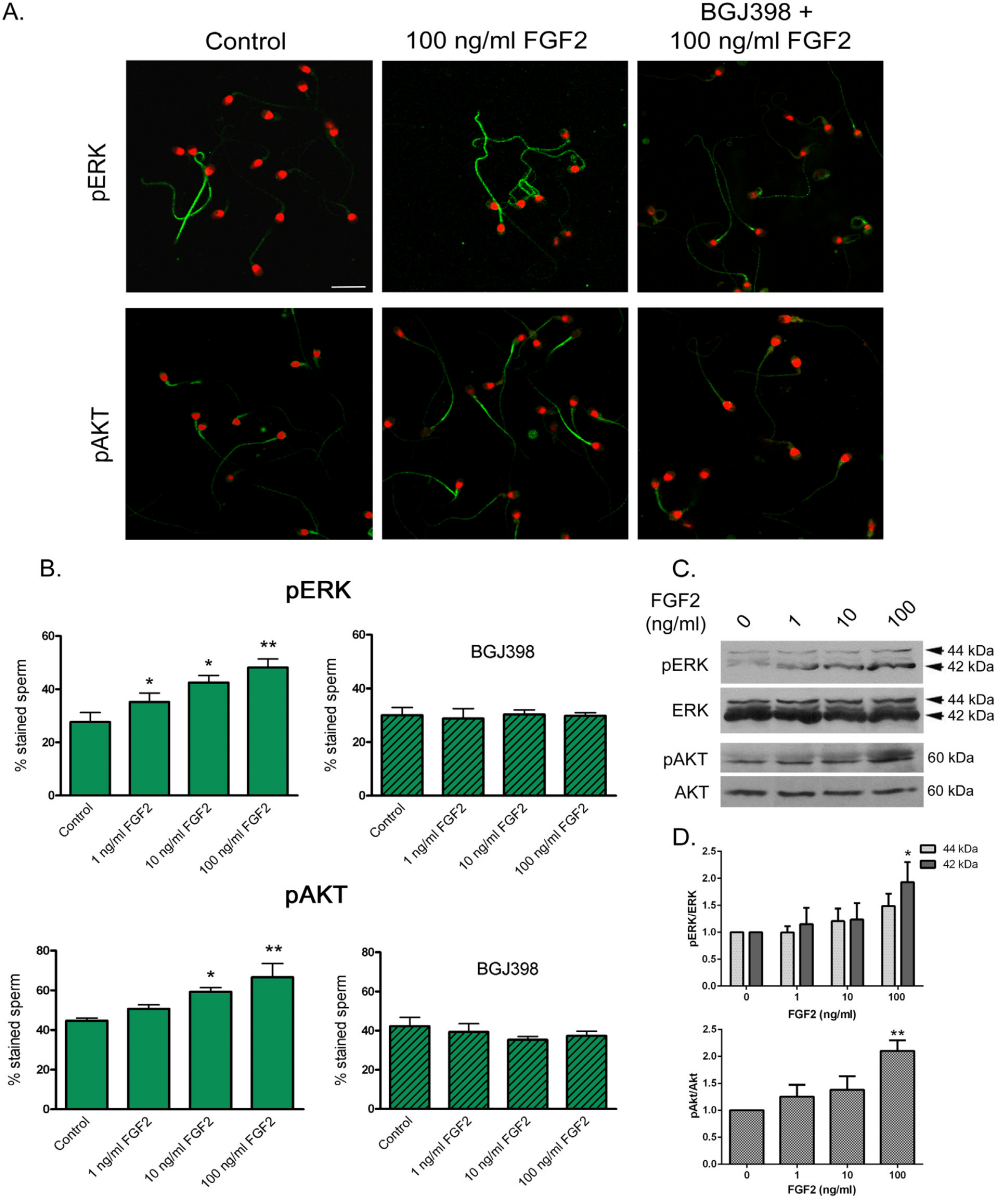
Activation of FGFR-related intracellular pathways in sperm exposed to FGF2. Sperm were incubated for a total 4-h period and exposed to FGF2 (0, 1, 10 and 100 ng/ml) for the last 15 min. In some aliquots, sperm were incubated for 15 min with BGJ398 (0.1 μM) before the addition of FGF2. (**A**) Immunolocalization of pERK and pAkt in sperm incubated in the absence of FGF2 (Control), with 100 ng/ml FGF2, and with BGJ398 + 100 ng/ml FGF2. Sperm were processed for immunocytochemistry, stained with anti pERK or pAkt and FITC-conjugated secondary antibodies; nuclei were stained with propidium iodide. Bar: 10 μm. (**B**) Percentage of sperm cells stained with anti pERK and anti pAkt after exposure to FGF2 in the absence (**left**) or presence of BGJ398 (**right**). Results are expressed as mean ± SEM, n = 4. * *P* < 0.05; ** *P* < 0.01 compared with Control. (**C**) Phosphorylation of ERK and Akt assessed by Western immunoblotting. Protein extracts from human sperm were subjected to SDS-PAGE and Western immunoblotting using anti pERK, ERK, pAkt and Akt antibodies. The estimated molecular weights of the protein bands are indicated on the right. (**D**) Densitometric analysis of Western immunoblotting results for pERK normalized to ERK and pAkt normalized to Akt. Results are expressed as mean ± SEM, n = 5 for ERK and n = 5 for Akt. * *P* < 0.05 and ** *P* < 0.01 compared with Control.

The activation of downstream components of FGFR-related intracellular pathways was also evaluated in sperm exposed to FGF2. Immunocytochemical studies using anti pERK and anti pAkt antibodies showed a significant increase (*P* < 0.05) in the percentage of cells stained in the flagellum in response to increasing concentrations of FGF2 (Fig 5A and 5B). These effects were blocked when sperm were preincubated with BGJ398 (Fig 5A and 5B). In agreement with these findings, Western immunoblot analysis revealed an increase in sperm pERK and pAkt signals after FGF2 exposure (Fig 5C and 5D).

### Effect of FGF2 upon human sperm motility

**Fig 6:**
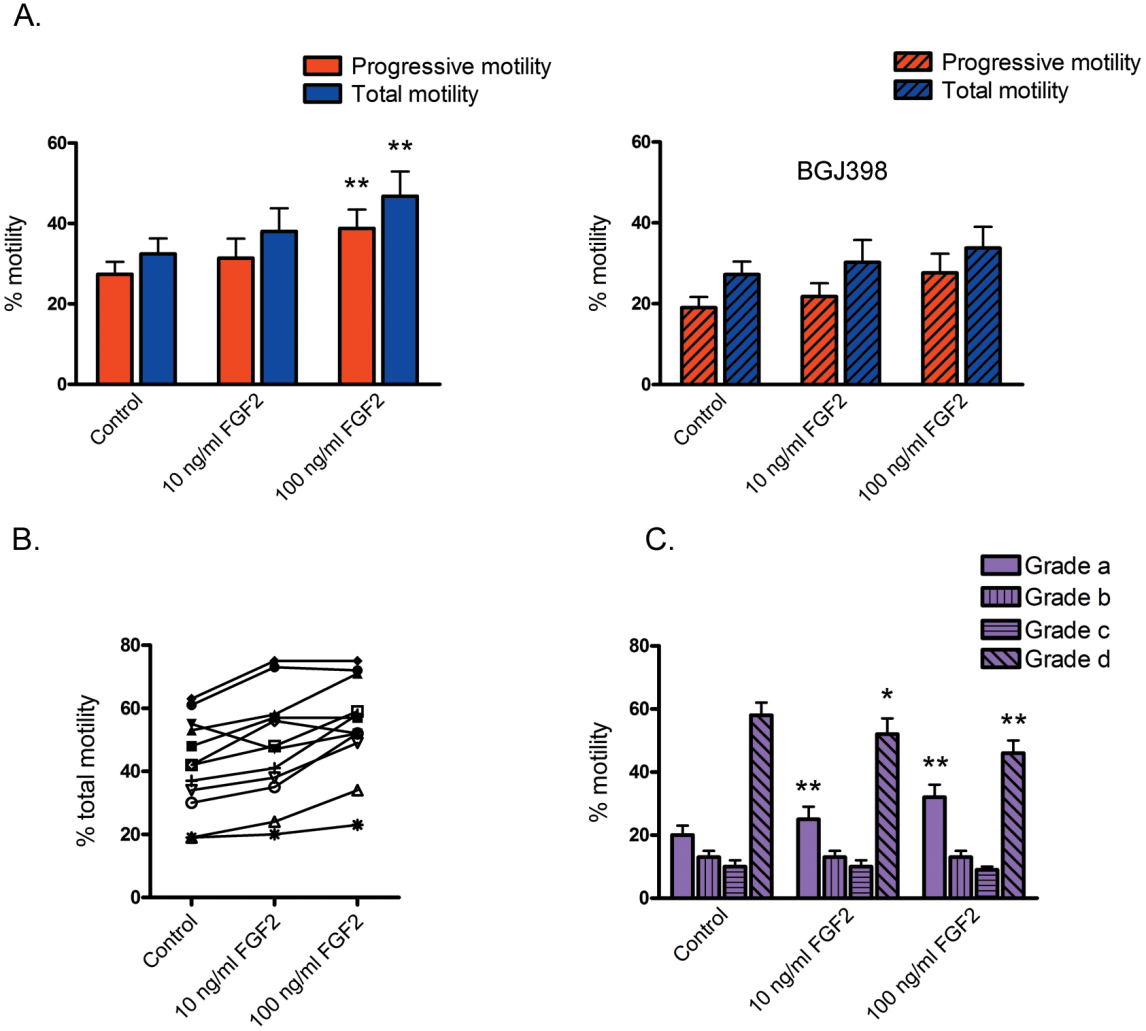
Effect of sperm incubation with FGF2 on sperm motility. Sperm were incubated with 0, 10 and 100 ng/ml FGF2 in the absence or in the presence of 0.1 μM BGJ398 and subjected to computer-assisted sperm analysis. (**A**) Percentages of progressive (Grade a + b) and total motility (Grade a + b + c) for aliquots incubated in the absence (**left**) or in the presence of BGJ398 (**right**). Results are expressed as mean ± SEM, n = 5. ** *P* < 0.01 compared with Control. (**B**) Individual recordings of the effect of sperm incubation with FGF2 (0, 10 and 100 ng/ml) on the percentage of total sperm motility in samples with low and high sperm motility. Each sample is identified with a different symbol (n = 12). (**C**) The percentages of sperm with Grade a, b, c and d motility in each condition (as defined in Materials and Methods) is depicted. * *P* < 0.05; ** *P* < 0.01 compared with the same Grade in Control (n = 12).

Sperm exposure to FGF2 resulted in an increase in the percentage of both progressive and total motility, reaching significance (*P* < 0.01) at 100 ng/ml FGF2. The effect was mediated by FGFR activation, since sperm preincubation with the inhibitor BGJ398 prevented such increase (Fig 6A).

The FGF2 effect upon motility was further assessed in a group of semen samples depicting a wide range of total sperm motility. After treatment with 10 and 100 ng/ml FGF2, the mean percentage of total sperm motility in these samples significantly increased in comparison with the Control (Control: 42 ± 4%, 10 ng/ml FGF2: 48 ± 5% (*P* < 0.05), and 100 ng/ml FGF2: 55 ± 4% (*P* < 0.01)). This effect was observed in samples with low (approximately 20%) and high percentages (approximately 60%) of total motile sperm (Fig 6B). In cells treated with 10 and 100 ng/ml FGF2 significantly higher percentages (*P* < 0.01) of Grade a motility were observed, concomitant with lower percentages (*P* < 0.05) of Grade d motility in comparison with the Control (Fig 6C). Additionally, incubation with 100 ng/ml FGF2 caused a significant increase (*P* < 0.01) in sperm kinematics, as reflected in higher values for most sperm motility parameters (curvilinear velocity (VCL), straight-line velocity (VSL), average path velocity (VAP), linearity (LIN), amplitude of lateral head displacement (ALH) and beat/cross frequency (BCF)) (Table 1).

**Table 1:** Kinematics of human sperm incubated with FGF2.

	Control	10 ng/ml FGF2	100 ng/ml FGF2
**VCL (μm/s)**	40 ± 6	45 ± 8	61 ± 9**
**VSL (μm/s)**	24 ± 4	28 ± 6	39 ± 6**
**VAP (μm/s)**	29 ± 5	32 ± 6	45 ± 7**
**LIN (%)**	39 ± 5	43 ± 7	58 ± 7**
**ALH (μm)**	2 ± 0	2 ± 0	3 ± 0**
**STR (%)**	54 ± 7	59 ± 9	78 ± 9
**BCF (Hz)**	4 ± 1	5 ± 1**	7 ± 1**

Computer-assisted sperm analysis of sperm incubated with 0, 10 or 100 ng/ml FGF2. Parameters measured were: curvilinear velocity (VCL), straight-line velocity (VSL), average path velocity (VAP), linearity (LIN), amplitude of lateral head displacement (ALH), straightness (STR), beat/cross frequency (BCF). Results are expressed as mean ± SEM, n = 12.

** *P* < 0.01 compared with Control.

## Discussion

The present study aimed to describe FGFR expression in the human testis, and to analyze their presence, localization, activation and function in the human sperm. Using primers designed to recognize sequence regions that are common to all splice variants of each FGFR and commercial antibodies directed against regions conserved in most isoforms, we were able to detect the expression of FGFR1, 2, 3 and 4 mRNA and protein forms in the human testis. The mRNA expression profiles of several FGFR splice variants (FGFR1 IIIb and IIIc, FGFR2 IIIc, FGFR3 IIIc and FGFR4) and of various FGF ligands have been thoroughly described in the fetal, immature and adult rat testis [28]. In the human testis, the expression of FGFR3 IIIc, but not IIIb, has been reported [29]; however further studies are needed to assess the expression of all the FGFR variants in this tissue.

Immunohistochemical analysis reported in the present investigation showed that FGFR1, 2, 3 and 4 localized to Sertoli and/or germ cells of the human seminiferous epithelium. In agreement with our findings, the presence of FGFR1 and FGFR3 in human spermatogonia has been previously described [29–31]. Nuclear translocation of FGFRs in response to the ligands has been found in various cell types, including fibroblasts, preadipocytes and Sertoli cell precursors [25, 32, 33]. In tumor cells, the interaction between FGFR2, the progesterone receptor and STAT5 in the nucleus after FGF2 and medroxyprogesterone acetate (MPA) treatment has been associated with increased gene transcription and cell proliferation [34]. Whether nuclear FGFRs act as germ cell transcription factors and participate in cell differentiation/proliferation during spermatogenesis remains to be elucidated.

To our knowledge, this study is the first one in describing the expression and localization of FGFRs in human sperm. Amplicons corresponding to FGFR1, FGFR3 and FGFR4 were found in human sperm by means of RT-PCR; however, under the conditions assayed, no FGFR2 transcript was detected. This result is in agreement with significantly reduced levels of FGFR2 mRNA found in germ cells in comparison with Sertoli and interstitial cells isolated from the mouse testis [18]. Protein forms corresponding to the 4 FGFRs were specifically detected in human sperm extracts, and similar isoforms were obtained in testicular protein extracts. Regarding FGFR1, a protein band of approximately 150 kDa was recognized, which would correspond to the complete protein form [35], also described in mouse and rat sperm using the same antibody [17]. Lower molecular weight bands were found with the anti FGFR1, which may be truncated forms or FGFR1 alternative splice variants [36]. The anti FGFR2 antibody led to the identification of a sperm band of approximately 125 kDa, similar to that reported in human thyroid carcinoma cells [37]. Anti FGFR3 revealed the mature and non-glycosylated forms of the receptor (approximately 128 and 101 kDa, respectively), as those described in human testis and chondrocytes [29, 38]. With anti FGFR4, a main protein band of 130 kDa was detected in sperm extracts. Immunocytochemical studies allowed the localization of FGFR1, 2, 3 and 4 in the flagellum and in the acrosomal region of ejaculated human sperm. Results of sperm flagellar localization of FGFR1, 2 and 4 are in agreement with their expression in the flagellum of elongating/elongated spermatids, suggesting the testicular origin of these sperm receptors. However, the presence of FGFR3 in ejaculated sperm but their lack of immunodetection in spermatids warrants further studies to clarify its tissue origin.

It has been shown that FGF2 interacts with several isoforms of FGFR1, 2, 3 and 4, inducing FGFR dimerization and phosphorylation [8]. In this study, to evaluate the sperm FGFR functionality, cells were exposed to FGF2 and FGFR phosphorylation was evaluated using a specific antibody against Tyr653/654 residues (phosphorylation sites conserved in the 4 receptors). FGF2 caused a significant increase in the phosphorylation of flagellar FGFRs; this response was blocked by preincubation with the inhibitor BGJ398 suggesting transphosphorylation of sperm FGFRs, as described in other cells types [39, 40].

Activation of FGF/FGFR signaling pathways in somatic cells has been shown to involve the activation of Ras/ERK and PI3K/Akt pathways [8]. Interestingly, some components of these pathways, such as ERK1/2, PI3K and Akt, have been described in mammalian sperm and have been shown to play an essential role in the maintenance of sperm function [41–44]. Our results showed an increase in both ERK and Akt phosphorylation in response to FGF2, at similar levels to those reported in sperm exposed to progesterone [45]. These effects seemed to be mediated through FGFR activation, since they were abolished in cells preincubated with BGJ398.

The flagellar localization of the phosphorylated FGFR, ERK and Akt proteins prompted us to analyze the participation of the FGF/FGFR system in the regulation of sperm motility. Samples incubated with 100 ng/ml FGF2 showed significantly higher percentages of total and progressive motility, as well as increased LIN, ALH, BCF and velocity parameters in comparison with control aliquots. FGF2 would mediate its effect on sperm motility by FGFR activation as preincubation with BGJ398 inhibited this response. These results clearly demonstrate a beneficial effect of FGF2 upon human sperm motility and are in line with a recent study reporting a trend towards motility improvement in frozen-thawed sperm incubated with FGF2 [46]. The enhancement in sperm motility obtained with FGF2 was comparable to that described for other compounds that stimulate the ERK signaling cascade [27] or that inhibit the phosphodiesterase activity [47]. It is worth mentioning that the FGF/FGFR system has been shown to facilitate somatic cell motility and migration by the activation of PI3K and ERK pathways [48, 49]. Future studies will help in further characterizing the participation of the FGF/FGFR system in the regulation of mammalian sperm motion and the underlying mechanisms.

FGF2 has been localized in the female reproductive tract, secreted by the oviduct epithelial cells, the oocyte and the *cumulus oophorus* cells [50, 51]. These reports describe the proliferative, mitogenic and angiogenic effect of FGF2 on somatic cells of the reproductive tissues, but do not address the effect of FGF2 present in the reproductive tract upon sperm physiology. Considering our findings and that several kinases of the FGF/FGFR signaling pathways (in particular ERK and PI3K/Akt) have been involved in the maintenance of sperm motility, capacitation, acrosomal exocytosis and survival [52, 53], we propose that FGF2 present in the endometrium, in the oviduct and in the oocyte vicinity could bind to sperm FGFRs and regulate those fertilization-related events.

In conclusion, our data demonstrate that FGFR1, 2, 3 and 4 are expressed in the human testis and sperm, localized to the acrosomal region and flagellum. Sperm FGFRs are functional, since exposure to FGF2 leads to their phosphorylation and the activation of intracellular pathways. In addition, incubation with FGF2 causes an increase in the percentage of motile cells and enhances sperm kinematics, suggesting that this system is involved in the regulation of sperm motility. Findings from the present study contribute to the understanding of sperm physiology and would have relevance in the diagnosis and/or treatment of subfertile patients. Moreover, characterization of FGF/FGFR signaling in sperm, a transcriptional- and traslational-inactive cell, would shed light on the functioning of this system and its non-genomic effects.

## Supporting Information

S1 Fig FGFR protein expression in MCF7 cells and immunoblotting assays to determine anti FGFR specificities. Protein extracts from MCF7 cells, human testis and sperm were subjected to SDS-PAGE and Western immunoblotting using anti FGFR antibodies preincubated or not with the corresponding blocking peptides, or rabbit IgG as control. The estimated molecular weights of the protein bands are indicated on the left.
(TIF)

S2 Fig Immunohistochemical assays to determine anti FGFR1, 2 and 3 specificities. Portions of human testis were fixed and processed for immunohistochemistry using anti FGFR antibodies preincubated with the respective blocking peptides. The specimens were counterstained with hematoxylin. Bar: 20 μm.
(TIF)

S3 Fig Immunocytochemical assays to determine anti FGFR1, 2 and 3 specificities. Sperm cells were stained with anti FGFR1, FGFR2 and FGFR3 preincubated with the respective blocking peptides and a secondary antibody labeled with Cy3. The corresponding fields stained with FITC-PSA to assess acrosomal status are shown on the right. Bar: 10 μm.
(TIF)

## References

[1] Ornitz DM, Itoh N (2001) Fibroblast growth factors. Genome Biol 2: REVIEWS3005 11276432 10.1186/gb-2001-2-3-reviews3005 PMC138918

[2] Yu PJ, Ferrari G, Galloway AC, Mignatti P, Pintucci G (2007) Basic fibroblast growth factor (FGF-2): the high molecular weight forms come of age. J Cell Biochem 100: 1100–1108. 17131363 10.1002/jcb.21116

[3] Givol D, Yayon A (1992) Complexity of FGF receptors: genetic basis for structural diversity and functional specificity. FASEB J 6: 3362–3369. 1464370

[4] Johnson DE, Williams LT (1993) Structural and functional diversity in the FGF receptor multigene family. Adv Cancer Res 60: 1–41. 8417497 10.1016/s0065-230x(08)60821-0

[5] Powers CJ, McLeskey SW, Wellstein A (2000) Fibroblast growth factors, their receptors and signaling. Endocr Relat Cancer 7: 165–197. 11021964 10.1677/erc.0.0070165

[6] Cotton LM, O'Bryan MK, Hinton BT (2008) Cellular signaling by fibroblast growth factors (FGFs) and their receptors (FGFRs) in male reproduction. Endocr Rev 29: 193–216. 10.1210/er.2007-0028 18216218 PMC2528845

[7] Mohammadi M, Olsen SK, Ibrahimi OA (2005) Structural basis for fibroblast growth factor receptor activation. Cytokine Growth Factor Rev 16: 107–137. 15863029 10.1016/j.cytogfr.2005.01.008

[8] Eswarakumar VP, Lax I, Schlessinger J (2005) Cellular signaling by fibroblast growth factor receptors. Cytokine Growth Factor Rev 16: 139–149. 15863030 10.1016/j.cytogfr.2005.01.001

[9] Hughes SE (1997) Differential expression of the fibroblast growth factor receptor (FGFR) multigene family in normal human adult tissues. J Histochem Cytochem 45: 1005–1019. 9212826 10.1177/002215549704500710

[10] Fon Tacer K, Bookout AL, Ding X, Kurosu H, John GB, Wang L, et al (2010) Research resource: Comprehensive expression atlas of the fibroblast growth factor system in adult mouse. Mol Endocrinol 24: 2050–2064. 10.1210/me.2010-0142 20667984 PMC2954642

[11] Turner N, Grose R (2010) Fibroblast growth factor signalling: from development to cancer. Nat Rev Cancer 10: 116–129. 10.1038/nrc2780 20094046

[12] Itoh N, Ornitz DM (2011) Fibroblast growth factors: from molecular evolution to roles in development, metabolism and disease. J Biochem 149: 121–130. 10.1093/jb/mvq121 20940169 PMC3106964

[13] Dorey K, Amaya E (2010) FGF signalling: diverse roles during early vertebrate embryogenesis. Development 137: 3731–3742. 10.1242/dev.037689 20978071 PMC3747497

[14] Chaves RN, de Matos MH, Buratini J Jr, de Figueiredo JR (2012) The fibroblast growth factor family: involvement in the regulation of folliculogenesis. Reprod Fertil Dev 24: 905–915. 10.1071/RD11318 22935151

[15] Jiang X, Skibba M, Zhang C, Tan Y, Xin Y, Qu Y (2013) The roles of fibroblast growth factors in the testicular development and tumor. J Diabetes Res 2013: 489095 10.1155/2013/489095 24159602 PMC3789391

[16] Xu B, Yang L, Hinton BT (2013) The role of fibroblast growth factor receptor substrate 2 (FRS2) in the regulation of two activity levels of the components of the extracellular signal-regulated kinase (ERK) pathway in the mouse epididymis. Biol Reprod 89: 1–13. 10.1095/biolreprod.112.107185 PMC4076368 23782834

[17] Cotton L, Gibbs GM, Sanchez-Partida LG, Morrison JR, de Kretser DM, O'Bryan MK (2006) FGFR-1 [corrected] signaling is involved in spermiogenesis and sperm capacitation. J Cell Sci 119: 75–84. 16352663 10.1242/jcs.02704

[18] Li S, Lan ZJ, Li X, Lin J, Lei Z (2014) Role of postnatal expression of fgfr1 and fgfr2 in testicular germ cells on spermatogenesis and fertility in mice. J Reprod Infertil 15: 122–33. 25202669 PMC4138418

[19] Giulianelli S, Cerliani JP, Lamb CA, Fabris VT, Bottino MC, Gorostiaga MA, et al (2008) Carcinoma-associated fibroblasts activate progesterone receptors and induce hormone independent mammary tumor growth: A role for the FGF-2/FGFR-2 axis. Int J Cancer 123: 2518–2531. 10.1002/ijc.23802 18767044

[20] Guagnano V, Furet P, Spanka C, Bordas V, Le Douget M, Stamm C, et al (2011) Discovery of 3-(2,6-dichloro-3,5-dimethoxy-phenyl)-1-{6-[4-(4-ethyl-piperazin-1-yl)-phenylamino]-pyrimidin-4-yl}-1-methyl-urea (NVPBGJ398), a potent and selective inhibitor of the fibroblast growth factor receptor family of receptor tyrosine kinase. J Med Chem 54: 7066–7083. 10.1021/jm2006222 21936542

[21] World Health Organization (2010) WHO Laboratory manual for the examination and processing of human semen, 5th edn. World Health Organization Press, Geneva, Switzerland.

[22] Lehtola L, Partanen J, Sistonen L, Korhonen J, Wärri A, Härkönen P, et al (1992) Analysis of tyrosine kinase mRNAs including four FGF receptor mRNAs expressed in MCF-7 breast-cancer cells. Int J Cancer 50: 598–603. 1311287 10.1002/ijc.2910500419

[23] Lapyckyj L, Castillo LF, Matos ML, Gabrielli NM, Lüthy IA, Vazquez-Levin MH (2010) Expression analysis of epithelial cadherin and related proteins in IBH-6 and IBH-4 human breast cancer cell lines. J Cell Physiol 222: 596–605. 10.1002/jcp.21974 19957299

[24] Marín-Briggiler CI, Veiga MF, Matos ML, Echeverría MF, Furlong LI, Vazquez-Levin MH (2008) Expression of epithelial cadherin in the human male reproductive tract and gametes and evidence of its participation in fertilization. Mol Hum Reprod 14: 561–571. 10.1093/molehr/gan053 18829448

[25] Widberg CH, Newell FS, Bachmann AW, Ramnoruth SN, Spelta MC, Whitehead JP, et al (2009) Fibroblast growth factor receptor 1 is a key regulator of early adipogenic events in human preadipocytes. Am J Physiol Endocrinol Metab 296: 121–131. 10.1152/ajpendo.90602.2008 18940940

[26] Marín-Briggiler CI, Lapyckyj L, González Echeverría MF, Rawe VY, Alvarez Sedó C, Vazquez-Levin MH (2010) Neural cadherin is expressed in human gametes and participates in sperm-oocyte interaction events. Int J Androl 33: e228–239. 10.1111/j.1365-2605.2009.00999.x 19840148

[27] Almog T, Lazar S, Reiss N, Etkovitz N, Milch E, Rahamim N, et al (2008) Identification of extracellular signal-regulated kinase 1/2 and p38 MAPK as regulators of human sperm motility and acrosome reaction and as predictors of poor spermatozoan quality. J Biol Chem 283: 14479–14489. 10.1074/jbc.M710492200 18372245

[28] Cancilla B, Davies A, Ford-Perriss M, Risbridger GP (2000) Discrete cell- and stage-specific localisation of fibroblast growth factors and receptor expression during testis development. J Endocrinol 164: 149–159. 10657850 10.1677/joe.0.1640149

[29] Ewen KA, Olesen IA, Winge SB, Nielsen AR, Nielsen JE, Graem N, et al (2013) Expression of FGFR3 during human testis development and in germ cell-derived tumours of young adults. Int J Dev Biol 57: 141–151. 10.1387/ijdb.130022er 23784824

[30] Steger K, Tetens F, Seitz J, Grothe C, Bergmann M (1998) Localization of fibroblast growth factor 2 (FGF-2) protein and the receptors FGFR 1–4 in normal human seminiferous epithelium. Histochem Cell Biol 110: 57–62. 9681690 10.1007/s004180050265

[31] von Kopylow K, Staege H, Schulze W, Will H, Kirchhoff C (2012) Fibroblast growth factor receptor 3 is highly expressed in rarely dividing human type A spermatogonia. Histochem Cell Biol 138: 759–772. 10.1007/s00418-012-0991-7 22777346

[32] Maher PA (1996) Nuclear Translocation of fibroblast growth factor (FGF) receptors in response to FGF-2. J Cell Biol 134: 529–536. 8707835 10.1083/jcb.134.2.529 PMC2120872

[33] Schmahl J, Kim Y, Colvin JS, Ornitz DM, Capel B (2004) Fgf9 induces proliferation and nuclear localization of FGFR2 in Sertoli precursors during male sex determination. Development 131: 3627–3636. 15229180 10.1242/dev.01239

[34] Cerliani JP, Guillardoy T, Giulianelli S, Vaque JP, Gutkind JS, Vanzulli SI, et al (2011) Interaction between FGFR-2, STAT5, and progesterone receptors in breast cancer. Cancer Res 71: 3720–3731. 10.1158/0008-5472.CAN-10-3074 21464042

[35] Dionne CA, Crumley G, Bellot F, Kaplow JM, Searfoss G, Ruta M, et al (1990) Cloning and expression of two distinct high-affinity receptors cross-reacting with acidic and basic fibroblast growth factors. EMBO J 9: 2685–2692. 1697263 10.1002/j.1460-2075.1990.tb07454.x PMC551973

[36] Groth C, Lardelli M (2002) The structure and function of vertebrate fibroblast growth factor receptor 1. Int J Dev Biol 46: 393–400. 12141425

[37] St Bernard R, Zheng L, Liu W, Winer D, Asa SL, Ezzat S (2005) Fibroblast growth factor receptors as molecular targets in thyroid carcinoma. Endocrinology 146: 1145–1153. 15564323 10.1210/en.2004-1134

[38] Yan D, Chen D, Cool SM, van Wijnen AJ, Mikecz K, Murphy G, et al (2011) Fibroblast growth factor receptor 1 is principally responsible for fibroblast growth factor 2-induced catabolic activities in human articular chondrocytes. Arthritis Res Ther 13: R130 10.1186/ar3441 21835001 PMC3239372

[39] Bellot F, Crumley G, Kaplow JM, Schlessinger J, Jaye M, Dionne CA (1991) Ligand-induced transphosphorylation between different FGF receptors. EMBO J 10: 2849–2854. 1655404 10.1002/j.1460-2075.1991.tb07834.x PMC452995

[40] Bae JH, Schlessinger J (2010) Asymmetric tyrosine kinase arrangements in activation or autophosphorylation of receptor tyrosine kinases. Mol Cells 29: 443–448. 10.1007/s10059-010-0080-5 20432069

[41] Luconi M, Barni T, Vannelli GB, Krausz C, Marra F, Benedetti PA, et al (1998) Extracellular signal-regulated kinases modulate capacitation of human spermatozoa. Biol Reprod 58: 1476–1489. 9623609 10.1095/biolreprod58.6.1476

[42] Luconi M, Marra F, Gandini L, Filimberti E, Lenzi A, Forti G, et al (2001) Phosphatidylinositol 3-kinase inhibition enhances human sperm motility. Hum Reprod 16: 1931–1937. 11527900 10.1093/humrep/16.9.1931

[43] de Lamirande E, Gagnon C (2002) The extracellular signal-regulated kinase (ERK) pathway is involved in human sperm function and modulated by the superoxide anion. Mol Hum Reprod 8: 124–135. 11818515 10.1093/molehr/8.2.124

[44] Koppers AJ, Mitchell LA, Wang P, Lin M, Aitken RJ (2011) Phosphoinositide 3-kinase signalling pathway involvement in a truncated apoptotic cascade associated with motility loss and oxidative DNA damage in human spermatozoa. Biochem J 436: 687–698. 10.1042/BJ20110114 21470189

[45] Sagare-Patil V, Galvankar M, Satiya M, Bhandari B, Gupta SK, Modi D (2012) Differential concentration and time dependent effects of progesterone on kinase activity, hyperactivation and acrosome reaction in human spermatozoa. Int J Androl 35: 633–644. 10.1111/j.1365-2605.2012.01291.x 22775762

[46] Sugihara K, Shibata TK, Takata K, Kimura T, Kanayama N, Williams R, et al (2013) Attenuation of fibroblast growth factor signaling by poly-N-acetyllactosamine type glycans. FEBS Lett 587: 3195–3201. 10.1016/j.febslet.2013.07.056 23968720 PMC4029356

[47] Tardif S, Madamidola OA, Brown SG, Frame L, Lefièvre L, Wyatt PG, et al (2014) Clinically relevant enhancement of human sperm motility using compounds with reported phosphodiesterase inhibitor activity. Hum Reprod 29: 2123–2135. 10.1093/humrep/deu196 25124668 PMC4481575

[48] Pintucci G, Moscatelli D, Saponara F, Biernacki PR, Baumann FG, Bizekis C, et al (2002) Lack of ERK activation and cell migration in FGF-2-deficient endothelial cells. FASEB J. 16: 598–600. 11919166 10.1096/fj.01-0815fje

[49] Kamura S, Matsumoto Y, Fukushi JI, Fujiwara T, Iida K, Okada Y, et al (2010) Basic fibroblast growth factor in the bone microenvironment enhances cell motility and invasion of Ewing's sarcoma family of tumours by activating the FGFR1-PI3K-Rac1 pathway. Br J Cancer 103: 370–381. 10.1038/sj.bjc.6605775 20606682 PMC2920026

[50] Gabler C, Lauer B, Einspanier A, Schams D, Einspanier R (1997) Detection of mRNA and immunoreactive proteins for acidic and basic fibroblast growth factor and expression of the fibroblast growth factor receptors in the bovine oviduct. J Reprod Fertil 109: 213–221. 9155730 10.1530/jrf.0.1090213

[51] Malamitsi-Puchner A, Sarandakou A, Baka SG, Tziotis J, Rizos D, Hassiakos D, et al (2001) Concentrations of angiogenic factors in follicular fluid and oocyte-cumulus complex culture medium from women undergoing in vitro fertilization: association with oocyte maturity and fertilization. Fertil Steril 76: 98–101. 11438326 10.1016/s0015-0282(01)01854-4

[52] de Lamirande E, O'Flaherty C (2008) Sperm activation: role of reactive oxygen species and kinases. Biochim Biophys Acta 1784: 106–115. 17920343 10.1016/j.bbapap.2007.08.024

[53] Breitbart H, Rotman T, Rubinstein S, Etkovitz N (2010) Role and regulation of PI3K in sperm capacitation and the acrosome reaction. Mol Cell Endocrinol 314: 234–238. 10.1016/j.mce.2009.06.009 19560510

