# Supplementary figures and images for: Fibroblast Growth Factor Receptors (FGFRs) in Human Sperm: Expression, Functionality and Involvement in Motility Regulation
Source: PLoS One. 2015 May 13;10(5):e0127297. doi: 10.1371/journal.pone.0127297 (PMC4430232; doi:10.1371/journal.pone.0127297)

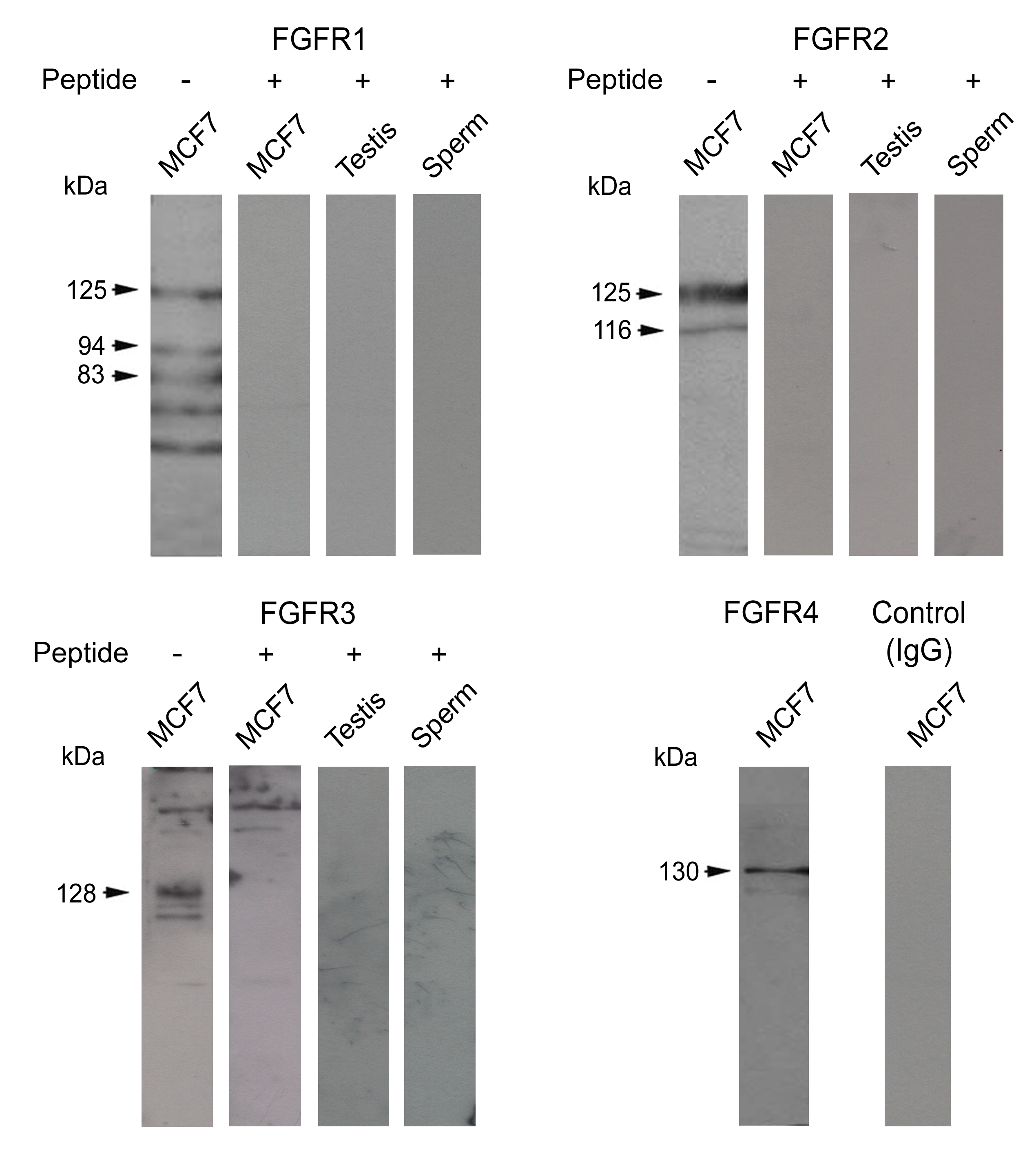

Supplement: S1 Fig — Protein extracts from MCF7 cells, human testis and sperm were subjected to SDS-PAGE and Western immunoblotting using anti FGFR antibodies preincubated or not with the corresponding blocking peptides, or rabbit IgG as control. The estimated molecular weights of the protein bands are indicated on the left. (TIF) [file pone.0127297.s001.tif]

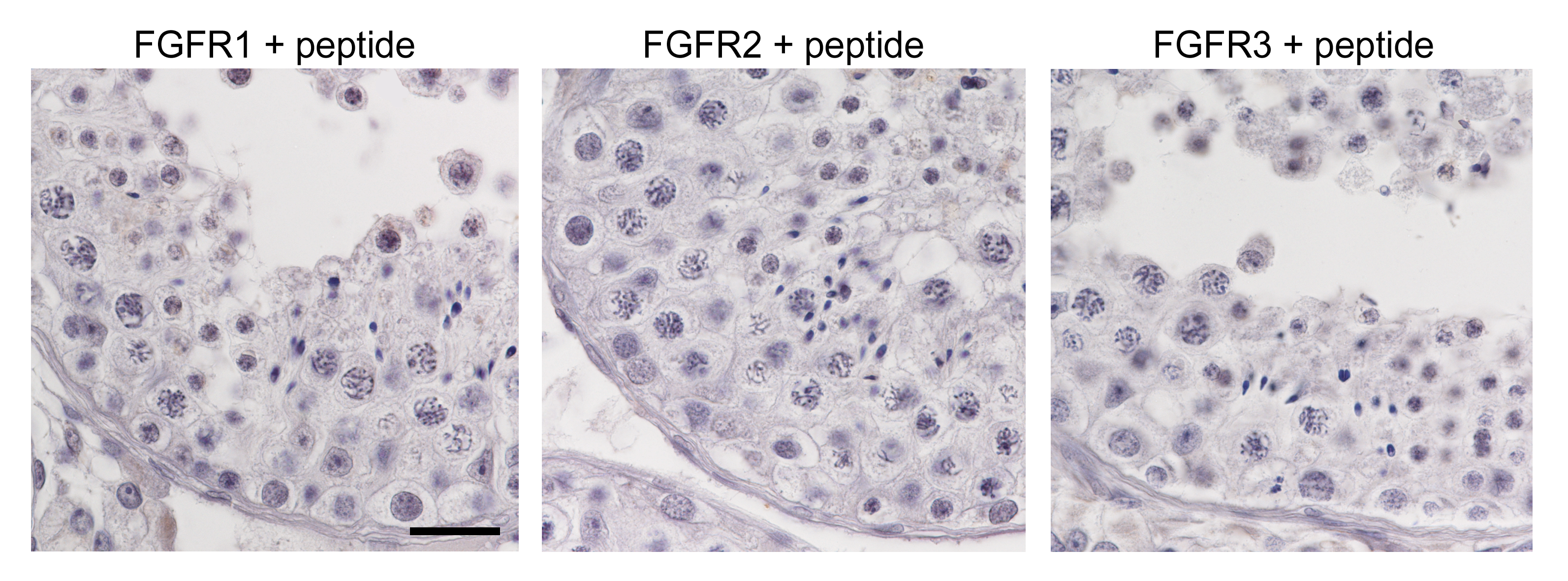

Supplement: S2 Fig — Portions of human testis were fixed and processed for immunohistochemistry using anti FGFR antibodies preincubated with the respective blocking peptides. The specimens were counterstained with hematoxylin. Bar: 20 μm. (TIF) [file pone.0127297.s002.tif]

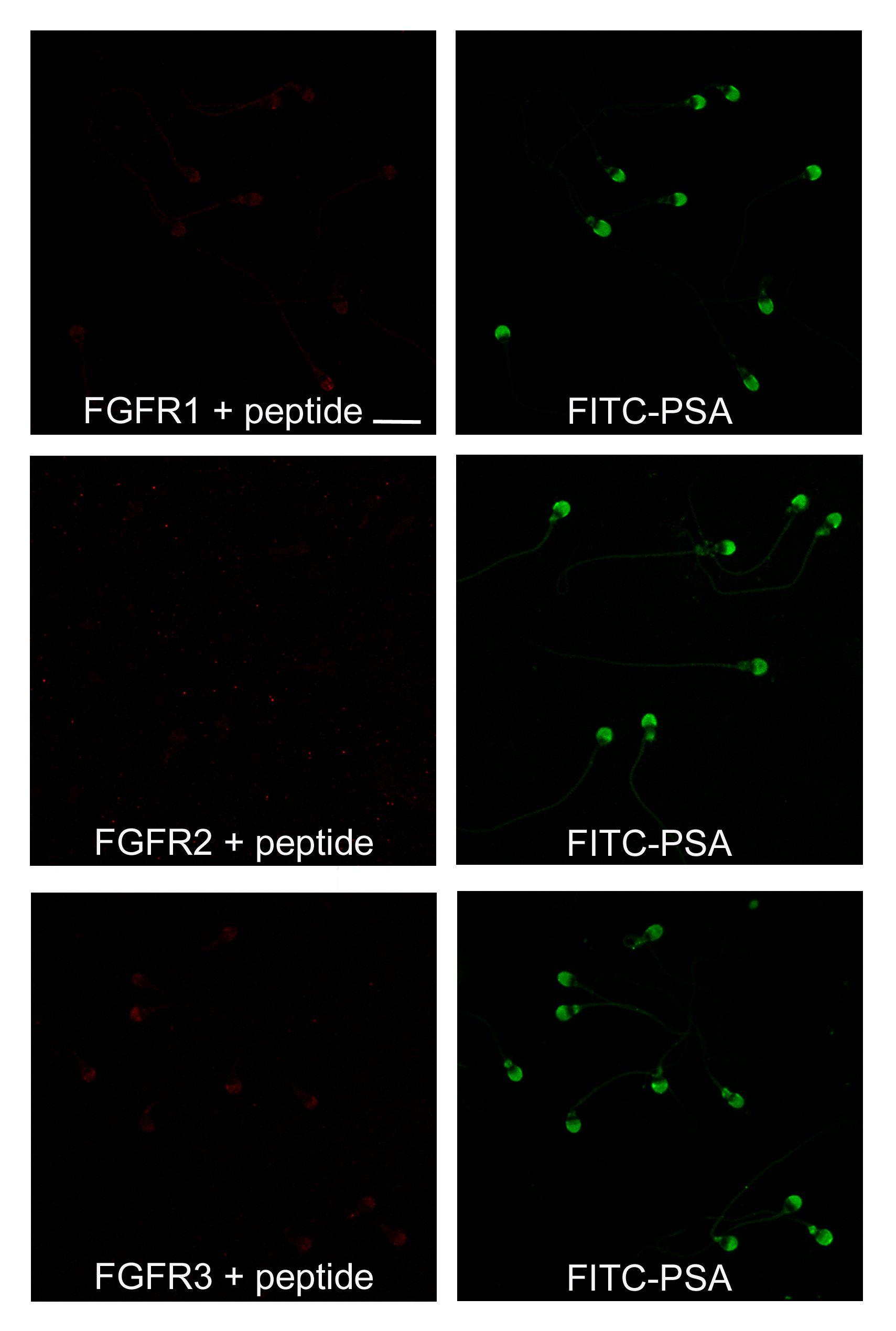

Supplement: S3 Fig — Sperm cells were stained with anti FGFR1, FGFR2 and FGFR3 preincubated with the respective blocking peptides and a secondary antibody labeled with Cy3. The corresponding fields stained with FITC-PSA to assess acrosomal status are shown on the right. Bar: 10 μm. (TIF) [file pone.0127297.s003.tif]
